# Assessing health systems for type 1 diabetes in sub-Saharan Africa: developing a 'Rapid Assessment Protocol for Insulin Access'

**DOI:** 10.1186/1472-6963-6-17

**Published:** 2006-02-24

**Authors:** David Beran, John S Yudkin, Maximilian de Courten

**Affiliations:** 1 International Insulin Foundation, International Health and Medical Education Centre, University College London, Holborn Union Building, Archway Campus, 2-10, Highgate Hill, London N19 5LW, UK

## Abstract

**Background:**

In order to improve the health of people with Type 1 diabetes in developing countries, a clear analysis of the constraints to insulin access and diabetes care is needed. We developed a Rapid Assessment Protocol for Insulin Access, comprising a series of questionnaires as well as a protocol for the gathering of other data through site visits, discussions, and document reviews.

**Methods:**

The Rapid Assessment Protocol for Insulin Access draws on the principles of Rapid Assessment Protocols which have been developed and implemented in several different areas. This protocol was adapted through a thorough literature review on diabetes, chronic condition management and medicine supply in developing countries.

A visit to three countries in sub-Saharan Africa and meetings with different experts in the field of diabetes helped refine the questionnaires. Following the development of the questionnaires these were tested with various people familiar with diabetes and/or healthcare in developing countries. The Protocol was piloted in Mozambique then refined and had two further iterations in Zambia and Mali. Translations of questionnaires were made into local languages when necessary, with back translation to ensure precision.

**Results:**

In each country the protocol was implemented in 3 areas – the capital city, a large urban centre and a predominantly rural area and their respective surroundings. Interviews were carried out by local teams trained on how to use the tool. Data was then collected and entered into a database for analysis.

**Conclusion:**

The Rapid Assessment Protocol for Insulin Access was developed to provide a situational analysis of Type 1 diabetes, in order to make recommendations to the national Ministries of Health and Diabetes Associations. It provided valuable information on patients' access to insulin, syringes, monitoring and care. It was thus able to sketch a picture of the health care system with regards to its ability to care for people with diabetes. In all countries where this tool was used the involvement of local stakeholders resulted in the process acting as a catalyst in bringing diabetes to the attention of the health authorities.

## Background

Leonard Thompson, a Canadian child, was given his first injection of insulin on 11 January 1922 [1]. He was the first patient to be treated with insulin for Type 1 diabetes. Having survived some 2 1/2 years from his diagnosis, he had done better than most Type 1 diabetic patients in the pre-insulin era. Three quarters of a century after its discovery, insulin is still not available on an uninterrupted basis in many parts of the developing world [2-4]. A survey in 25 countries in Africa found that in half of them insulin was often unavailable in the large city hospitals, while in only 5 countries was insulin regularly available in rural areas. In some countries, insulin is not included on the national formulary [5]. The International Diabetes Federation estimates that there are 35,100 children with Type 1 diabetes in Africa [6].

Before the discovery and purification of insulin, the average life expectancy for a patient with Type 1 diabetes was around 12 months [7]. Some studies suggest that the prognosis for a patient with Type 1 diabetes in sub-Saharan Africa may currently be just as poor [8-10]. The reasons for this are that patients with Type 1 diabetes need a continuing supply of insulin, syringes and monitoring, and a health care system able to provide these supplies to all parts of the country. In parallel to these supplies diabetes-trained health workers able to provide both clinical support and patient education are also essential. There have been no systematic studies in countries in sub-Saharan Africa of health care for patients with Type 1 diabetes.

In order to study such patterns, a clear analysis of the constraints to insulin access and diabetes care is needed. It is likely that increasing the supply of insulin through donations or other means is not sustainable and the root of the problems needs to be solved. This led to the development of the Rapid Assessment Protocol for Insulin Access (RAPIA).

The framework of the RAPIA studies the path of insulin from its arrival in the country to the point that it reaches or fails to treat the patient effectively and thereby identify how and where the system works and/or fails. The aim of this process is to look at the provision of care for Type 1 diabetes from the viewpoint of the entire health system to see where improvements are necessary to address the problems that hamper access to insulin and care for their diabetes.

## Methods

The RAPIA draws on the principles of Rapid Assessment Protocols (RAP) which have been developed and implemented in different areas. The method of RAP has been used extensively to assess services for communicable diseases, including malaria, tuberculosis and STDs, for the purpose of developing interventions. The approach chosen here is to adapt existing protocols to suit the assessment of access to insulin [11-13].

The main principles of the RAP are:[14]

- Pragmatism – the methods should provide adequate information, without necessarily being 'scientifically perfect'. Triangulation, or cross-checking between different sources of data is used to establish the validity and reliability of the data collected.

- Speed – the methods are intended to provide relevant information quickly, upon which decisions about health care interventions can be made.

- Use of multiple data sources – different methods are used to access different sources of data to get a balanced overview.

- Cost-effectiveness – the focus is on research instruments that provide information cheaply, and are not labour and time intensive. Where possible, use is made of existing data.

These were adapted through a thorough literature review on diabetes, chronic condition management and medicine supply in developing countries. This literature review was carried out using Medline.

Based on these articles and discussions with different experts in the field a conceptual model was developed. This model comprised two paths, the path of insulin and the path of care.

All the stakeholders at different levels of the health system within a country were listed that would have an influence on these two factors. These two paths then served as the basis for the development of the questionnaires. Different questionnaires targeted at different stakeholders had overlapping questions in order to check for internal consistency.

Once the draft version of the RAPIA was ready a visit to Mozambique, Zambia and Tanzania, with meetings at the Ministry of Health, in hospitals, with diabetes associations, clinicians and patients enabled the lead author to discuss the problems each person had with providing/obtaining care and insulin. This also enabled some questions to be tested and explore the issues surrounding diabetes care and insulin access in more depth in the field.

The information gathered from these visits enabled the final development of the questionnaires and protocol. Following the development of the questionnaires these were tested with various people familiar with diabetes and/or healthcare in developing countries.

The aim of the RAPIA is to investigate the possible barriers to insulin access that exist in a particular country. The framework of the RAPIA studies the path of insulin from its arrival in the country to the point that it reaches or fails to treat the patient effectively and thereby identify how and where the system works and/or fails. The questionnaires developed and the questions included in them tried to follow each level of this path from the beginning with the purchase and import of insulin to the country until the insulin reached or failed to reach the patient. In parallel the path of care was assessed from the highest levels of the health system, to regional/provincial organisations, hospitals, clinics and finally individual carers and patients.

The questionnaires developed serve as a guide and can be adapted according to the structure of the country. In order to achieve the broad aim, the RAPIA has the following objectives:

- To provide a range of data collection tools, from which research teams can select those appropriate to their own situation

- To provide suggestions of data items to collect

- To provide suggestions on data sources, data collection, data analysis and data presentation for each of the tools presented.

- To collect opinions and perspectives of the different people interviewed rather than precise statistical data.

The RAPIA provides a practical field guide to assist teams in the collection, analysis and presentation of data to evaluate and inform the development of health care services for diabetes management in resource poor settings. It provides the tools to enable a research team to collect information on the structure and functioning of insulin supply services/practices and also to conduct an assessment of the quality of care currently provided to people with Type 1 diabetes. This information is gathered through the different questionnaires detailed in Table 1, site visits, document reviews and discussions. These discussions were usually held informally regarding a specific problem or issue seen during an interview, site visit or document review. They usually served the purpose of clarifying certain aspects or getting a further insight into a specific issue in a less formal way.

The RAPIA provides information in the categories of:

- Health service structure and functioning with regards to procurement of medicines, diabetes management

- Diabetes policies written and enacted

- Reported and observed practice for diabetes management

- Availability of insulin, syringes and monitoring equipment

- Price of insulin, syringes and monitoring equipment

- Existence of distribution networks for insulin

- Insulin supply-related knowledge and attitudes amongst people with diabetes and their carers.

- Other problems that hamper the access to proper insulin and care

Table 2 shows the specific areas that the different RAPIA questionnaires target.

The RAPIA is divided into 3 components (Table 1). The first is the Macro level which is aimed at the Ministerial levels, Private Sector, National Diabetes Association, Central Medical Store and Educators. Then the Meso level targeted at Provincial Health Officers, "Health Care Settings" (Hospitals, Clinics, Health Centres, etc.) and Pharmacies/Dispensaries. Finally a Micro level where Carers (Healthcare Workers and Traditional Healers) and people with diabetes are interviewed.

In each country the Meso and Micro levels of the protocol were implemented in 3 areas – the capital city, a large urban centre and a predominantly rural area and their respective surroundings. These three areas were chosen by local stakeholders to be representative of different geographical and socio-economic situations within the country. Local teams trained on how to use the tool carried out interviews.

For each of the Macro and Meso the aim was to collect all the necessary information. This meant that sometimes one questionnaire was answered by many people. For the Educators 4 were chosen from both medical and nursing faculties. In each area based on local discussions a representative sample of healthcare centres, hospitals and private facilities were chosen. The aim was to cover both different geographic and socio-economic sectors of a given area. For laboratories and pharmacies these questionnaires were carried out at each facility visited and also in the private sector.

For the Micro level the focal point for diabetes care in the area was interviewed as well as any healthcare worker working with them. At each facility visited at least one person was interviewed. This person was chosen based on the fact that they would be the most likely person to treat a person with diabetes. For traditional healers the main areas/markets where these practictioners were present were targeted. Local traditional experts were also identified through discussions with patients and these were also interviewed.

With regards to patients, these were identified either through the diabetes association or during clinic days. Again a variety of patients were interviewed, the aim being to reach theoretical saturation, i.e. those interviewed reported having the same problems as the others interviewed.

After the pilot of the RAPIA in Mozambique a guideline number of interviews was established. These are detailed in Table 3. These numbers are only to serve as a guide, the aim being to collect as much data as possible and also to investigate any specific problems and their reasons during this process.

Data was then collected and entered into a database for analysis.

Local stakeholders were identified through initial contacts with diabetes associations and the Ministries of health in these respective countries.

Besides the collection of data the RAPIA also identified areas for improvement and ways of going improving the current situation.

A copy of the RAPIA protocol is available from the lead author.

## Results

The Protocol was piloted in Mozambique and then refined with two further iterations in Zambia and Mali. Translations of questionnaires were made into local languages when necessary, with back translation to ensure precision.

Mali, Mozambique and Zambia were chosen due to their geographical, historical and socio-economic differences. Implementing the RAPIA in these three Highly Indebted Poor Countries (HIPCs) was to see how a sustainable solution could be found to the issues of access to insulin and proper diabetes care under extreme conditions of scarce resources in the health sector.

Each interview had as its main aim to obtain the person's perspective on the problems faced by people with diabetes in the given country in gaining access to insulin and proper diabetes care, rather than seeking precise statistical information. Through the overlap in the areas of questioning above and by using other sources of information (site visits, discussions and document reviews) the information gathered can be cross-checked and triangulated between different sources and types of information. These different interactions also allowed for new sources of information to be identified and used accordingly.

### The RAPIA process

**Figure 1:**
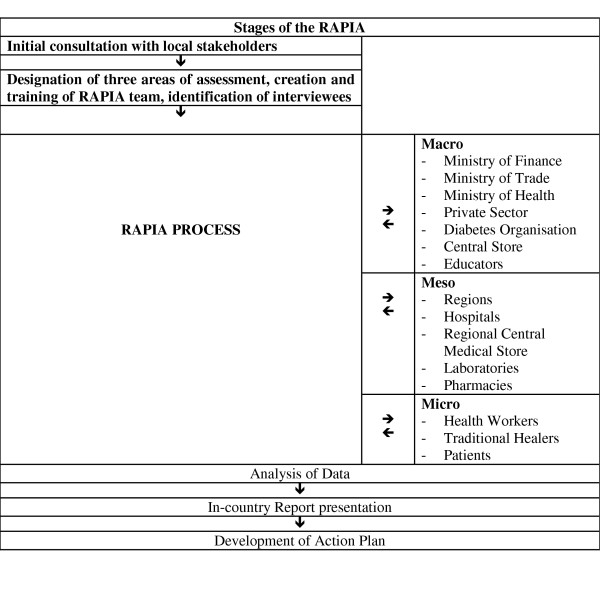
Stages of the RAPIA.

In each country the following steps described in Figure 1 were carried out to ensure a successful project.

Mozambique was chosen as a pilot country due to strong local support. DB together with a team of local interviewers from the diabetes association carried out the RAPIA in Mozambique over a period of almost 2 months. In collaboration with the Diabetes Association of Zambia (DAZ) and the support of the Central Board of Health, DB carried out the RAPIA in Zambia with a team of local interviewers, patients and carers from the diabetes association, over a period of a month. Together with Santé Diabète Mali (SDM), a local NGO, and the support of the Ministry of Health, and the Direction Nationale de la Santé, Dr. A. Nientao, DB carried out the RAPIA in Mali in 7 weeks. The number of interviews carried out in the different countries is detailed in Table 4.

Results were obtained in these three countries on the following:

- Estimates of prevalence for diabetes in different areas of the country. These were calculated using patient registers, interviews with healthcare workers and quantities of insulin ordered

- Estimates of life expectancy in different areas of the country. This was calculated using the prevalence determined above, with the incidence of 1 per 100,000 [6] used by the International Diabetes Federation (IDF) and the formula Prevalence = Incidence × Life Expectancy

- Purchase procedures, pricing, distribution, supply and availability of insulin throughout the country

- Purchase procedures, pricing, distribution, supply and availability of syringes throughout the country

- Levels of care in different facilities with regards to diabetes care

- Availability and cost of diagnostics

- Levels of healthcare worker training

- Role and activities of the diabetes associations

- Policies regarding diabetes

- Role of traditional healers

- Other barriers to care and insulin, for example travel, distance and cost

This data enabled the preparation of three country specific reports (available on ) highlighting the problems identified during the RAPIA and proposing concrete actions to address them. These reports were presented to local stakeholders and with the input of external experts and the IIF clear Action Plans were developed. A more comprehensive review of the data from Mozambique and Zambia have been published elsewhere [10].

### Limitations

As with any methodology certain limitations were present. These are:

- Representativeness of the 3 areas chosen in each country

- The sample was not random as a convenience sample was used

- Open questions used in questionnaires

- Questions with regards to its generalisability between the different areas and different countries

- Use of different interviewers in different areas

- Many of the interviewees chosen by local partners

- Patients interviewed lucky/fortunate ones – as they are the ones that are members of diabetes associations or have been able to attend a facility where there is some record of them

Out of the above list the key limitation is the non-random selection of areas, participants and stakeholders. This is due to the need for timely implementation and result generation and also due to the more qualitative nature of the approach (as opposed to a quantitative survey).

Careful identification with local input is the key to minimise selection bias. Triangulation is then rather the tool to verify the information gathered, which can to some extent also alert to non-representative (non-confirmed) findings, as it compares data gathered through different means to verify its validity.

Open questions are rather a strength in exploratory research. Generalisability is limited, but the patterns observed and frameworks for implementing solutions are comparable across settings and this is again a strength of our approach. The end product (country implementation plan) however will be locally anchored and relevant rather than internationally applicable.

## Conclusion

Besides providing valuable information on patients' access to insulin, syringes, monitoring and care which was then used to help these three countries improve the care for people with diabetes, the RAPIA was able to sketch a picture of the health care system and its ability to treat patients with Type 1 diabetes. In all countries where this tool was used the involvement of local stakeholders resulted in the process acting as a catalyst in bringing diabetes to the attention of the health authorities. The RAPIA also raised the profile of diabetes associations and awareness around diabetes and non-communicable diseases (NCDs) in countries where most funding and projects are focused on communicable diseases.

It has been proposed that illnesses such as Type 1 diabetes may represent a 'tracer' condition for effective health care systems [15,16], such that the management of patients with Type 1 diabetes could act as a yardstick against which the components of a fully functioning and effective health care system might be judged. A system with such components – including continuing supplies of drugs, diagnostic facilities, health worker training and retention, and patient education – are vital in the management of other NCDs and of the chronic communicable diseases such as tuberculosis and HIV/AIDS. Improvements in health care systems are, then, a vital component of improving health and health care across sub-Saharan Africa. Therefore the RAPIA besides enabling health planners to assess their health system with regards Type 1 diabetes could use this tool for an assessment of the capacity of their health system to deal with all chronic conditions.

It is intended that the RAPIA be freely available for use by health system planners, in Ministries, NGOs and international organisations, in other countries in Africa and elsewhere. The latest version of the RAPIA is available from the lead author, and discussions are proceeding with WHO divisions in Geneva and in Africa, as well as with the IDF, to disseminate the tool more widely for assessing health services for diabetic patients in resource poor countries.

## Abbreviations

DAZ – Diabetes Association of Zambia

HIPCs – Highly Indebted Poor Countries

IDDM – Insulin Dependent Diabetes Mellitus

IDF – International Diabetes Federation

IIF – International Insulin Foundation

NCDs – Non Communicable Diseases

RAP – Rapid Assessment Protocol

RAPIA – Rapid Assessment Protocol for Insulin Access

SDM – Santé Diabète Mali

WHO – World Health Organization

## Competing interests

The author(s) declare that they have no competing interests.

## Authors' contributions

DB drafted the manuscript, edited and revised the contents, MdC and JY edited and revised and reviewed the manuscript, DB, MdC and JY all contributed to the conceptual development of the project.

## Pre-publication history

The pre-publication history for this paper can be accessed here:



## Figures and Tables

**Table 1:** Questionnaires that make up the RAPIA

**Level**	**Issues Addressed in each RAPIA questionnaire**
	**MACRO**
- Ministry of Health	- Organisation of delivery of diabetes care - Resources available for diabetes and insulin - National Programs for diabetes and insulin - Pricing of insulin - Distribution of insulin - Funding for insulin and diabetes - Insulin tendering and purchase
- Ministry of Trade	- Trade issues (laws, barriers to trade) - Trade infrastructure
- Ministry of Finance	- Funding of health system - Taxes on insulin - Funding for insulin and diabetes
- Private Sector	- Pricing of insulin - Distribution of insulin
- National Diabetes Association	- Issues with diabetes and insulin
- Central Medical Store	- Insulin tendering and purchase - Insulin distribution and storage - Insulin pricing
	**MESO**
- Regional/District Health Organisation	- Issues with diabetes and insulin - Organisation of care for people with diabetes
- Hospitals, Clinics, Health Centres, Dispensaries, etc.	- Treatment and management of people with diabetes - Access to appropriate tools to diagnose and treat patients - Infrastructure present and/or lacking for insulin provision
- Laboratory	- Infrastructure present and/or lacking for proper diagnosis and follow-up
- Pharmacy	- Insulin distribution and storage - Insulin pricing
	**MICRO**
- Health Workers and Traditional Healers	- Problems encountered in diagnosis and treatment of patients - Training - Infrastructure present and/or lacking - Tools present and/or lacking
- Patients	- Diagnosis - Access to treatment - Cost of treatment

Taken from [10]

**Table 2:** Target areas of each RAPIA questionnaire

**Target questions with regards to insulin and diabetes care**	**QUESTIONNAIRES**														
	
	**MACRO**							**MESO**					**MICRO**		
	
	**A**	**B**	**C**	**D**	**E**	**F**	**G**	**H**	**I**	**J**	**K**	**L**	**M**	**N**	**O**
Funding for diabetes care and insulin		X	X			X		X	X	X					X
Taxes and Import restrictions on insulin	X	X	X	X	X	X			X			X			
Labour Resources			X					X		X					
Organisation of care			X		X			X		X			X	X	X
Supply of insulin and related supplies (cost, mark-ups, taxes, "black market")	X		X	X	X	X		X	X	X	X	X	X		X
Infrastructure			X	X		X		X	X	X	X	X	X		
Tools for monitoring and administration diabetes care			X		X			X		X	X		X		X
Training			X		X		X						X	X	
Awareness and education			X		X		X				X	X	X	X	X
Prevalence, Incidence number of cases seen			X		X		X	X		X	X	X	X	X	
Process of care (from diagnosis to treatment)			X		X			X		X	X	X	X	X	X

**Macro **– A (Ministry of Trade), B (Ministry of Finance), C (Ministry of Health), D (Private Sector), E (National Diabetes Association), F (Central Medical Store), G (Educators)

**Meso **– H (Regional Health Organisation), I (Regional Central Medical Store), J (Hospitals, Clinics, Health Centres, etc.), K (Laboratories), L (Pharmacies),

**Micro **– M (Health Workers), N (Traditional Healers), O (Patients)

**Table 3:** Guideline number of interviews for the RAPIA

	**Capital City**	**Urban Area**	**Rural Area**
Hospitals and Health Centres (Public and Private)	8	6	4
Pharmacies	8	6	4
Laboratories	8	6	4
Healthcare workers	20	10	5
Traditional Healers	8	6	4
Patients	20	10	5

**Table 4:** Number of interviews and discussions for RAPIAs in Mali, Mozambique and Zambia

**Type of interview/discussion**	**Mali**	**Mozambique**	**Zambia**	**Total**
**Patient**	39	20	38	**97**
**Pharmacy**	20	7	25	**52**
**Healthcare workers**	24	25	60	**109**
**Traditional Healers**	20	5	19	**44**
**Health Facilities**	16	13	22	**51**
**Regional level**	11	8	4	**23**
**Other**	20	27	49	**96**
**Total**	**150**	**105**	**217**	**472**

## References

[B1] Burrow G, Hazlett BE, Phillips MJ (1982). A case of diabetes mellitus. N Engl J Med.

[B2] McLarty D, Swai ABM, Alberti KGMM (1994). Insulin availability in Africa: an insoluble problem?. International Diabetes Digest.

[B3] Savage A (1994). The Insulin dilemma: a survey of Insulin treatment in the tropics. International Diabetes Digest.

[B4] Deeb L, Tan MH, Alberti KGMM (1994). Insulin availability among International Diabetes Federation member associations. Diabetes Care.

[B5] Alberti KGMM (1994). Insulin: availability and cost. World Health Forum.

[B6] Gan D, International Diabetes Federation (2003). Diabetes Atlas.

[B7] Bliss M (1997). The discovery of insulin: the inside story. Publ Am Inst Hist Pharm.

[B8] Castle W, Wicks A (1980). A follow-up of 93 newly diagnosed African diabetics for 6 years. Diabetologia.

[B9] Yudkin JS (2000). Insulin for the world's poorest countries. Lancet.

[B10] Beran D, Yudkin JS, de Courten M (2005). Access to care for patients with insulin-requiring diabetes in developing countries: Case studies of Mozambique and Zambia. Diabetes Care.

[B11] Manderson L, Aaby P (1992). An epidemic in the field? Rapid assessment procedures and health research. Social Science and Medicine.

[B12] Scrimshaw SCM, Hurtado E (1997). Rapid assessment procedures for nutrition and primary health care. Anthropological approaches to improving programme effectiveness.

[B13] Rhodes T, Stimson GV, Fitch C, Ball A, Renton A (1999). Rapid assessment, injecting drug use, and public health. Lancet.

[B14] World Health Organization (2002). SEX-RAR guide: the rapid assessment and response guide on psychoactive substance use and sexual risk behaviour.

[B15] Kessner DM, Carolyn EK, Singer J (1973). Assessing health quality: the case for tracers. N Engl J Med.

[B16] Hopkinson B, Balabanova D, McKee M, Kutzin J (2004). The human perspective on health care reform: coping with diabetes in Kyrgyzstan. Int J Health Plann Manage.

